# Addressing Motivation in Globesity Treatment: A New Challenge for Clinical Psychology

**DOI:** 10.3389/fpsyg.2012.00317

**Published:** 2012-09-03

**Authors:** Giada Pietrabissa, Gian Mauro Manzoni, Stefania Corti, Nadia Vegliante, Enrico Molinari, Gianluca Castelnuovo

**Affiliations:** ^1^Psychology Research Laboratory, Istituto Auxologico Italiano IRCCS, Ospedale San GiuseppeVerbania, Italy; ^2^Department of Psychology, Catholic University of MilanMilan, Italy; ^3^Department of Psychology, University of BergamoBergamo, Italy

The global epidemic of obesity and being overweight (Globesity) is rapidly becoming a major public health problem in many parts of the world, since it is often associated with many related chronic diseases (Wadden et al., 2002) including diabetes mellitus, cardiovascular disease, hypertension, kidney failure, osteoarthritis as well as psychological problems such as depression, feelings of shame, low self-esteem, and stigma (Byrne et al., 2004; Riva et al., 2006a; Manzoni et al., 2008; Villa et al., 2009; Capodaglio et al., 2010). Obesity and being overweight are also frequently connected with Binge Eating Disorder (BED), a psychopathological disorder characterized by frequent and persistent episodes of binge eating characterized by loss of control over eating within a discrete period of time, and significant distress.

In the United States, where obesity has become an epidemic over the past 20 years, 79% of the population is worryingly overweight. This means that only about 21 of 100 Americans are at or below normal weight (Flegal et al., 2010). Unfortunately, the problem of expanding waistlines does not just belong to the USA anymore. Mexico, for example, with 24.2% of the adult population classified as obese, is second to the USA in this trend, and Europe is currently competing with the North American countries for first place in the obesity crisis. In fact the United Kingdom reports 23% of their population to be clinically obese and predicts an increase in future years (Rennie and Jebb, 2005). There are also significant health care costs associated with treating obesity and its related diseases. Additionally, many people are unemployed as a direct result of over weightness, either on health grounds or due to other circumstances, leading to discrimination, stigma, and social exclusion (McCormick and Stone, 2007).

The causes of obesity involve a complex interplay of a number of factors, such as poor diet, lack of physical activity, certain medical disorders, and a range of well-documented genetic and environmental elements. Because the etiology of obesity is largely multifactorial, effective interventions to significantly reduce weight, maintain weight loss, and reduce related pathologies are typically combined treatment options (dietetic, nutritional, physical, behavioral, psychological, pharmacological, surgical). Important difficulties in these kinds of approaches, however, have been underlined with regard to availability, costs, treatment adherence, and long-term efficacy (Castelnuovo et al., 2003a, 2004a, 2010, 2011b,c,d; Gaggioli et al., 2003, 2005; Riva et al., 2003, 2004, 2006b; Manzoni et al., 2008, 2011; Castelnuovo, 2010a,b; Castelnuovo and Simpson, 2011). In fact, most overweight and obese individuals regain about one third of the weight lost with treatment within 1 year and they will typically return to baseline in 3–5 years (Castelnuovo et al., 2011d).

In addition, despite the dramatic increase in the prevalence of being overweight or obese, this does not reflect an equally proportionate expansion of the continuing education and training provided for health care professionals, across disciplines. Too often, health care professionals demonstrate a poor understanding and lack of recognition of the social and environmental determinants of obesity, simply suggesting that individuals should follow a diet and assuming that healthcare interventions will only be required when obesity-related medical complications are apparent. Obesity treatment should be individually tailored, and realistic goals should be clearly set at the outset. Psychological factors, in particular, influence both weight loss and, more importantly, long-term weight loss maintenance. Indeed, many recent studies (Svensson and Lagerros, 2010; Pettersen et al., 2011; Roberts and Bailey, 2011; Teufel et al., 2011; Walpole et al., 2011; West et al., 2011; Yilmaz et al., 2011; Stotland et al., 2012; Waller, 2012) have shown the crucial role of motivation in the realm of lifestyle modification, which has in turn inspired the development of a range of motivational enhancement techniques. In fact, although existing weight maintenance programs emphasize both behavioral and cognitive skill refinement, it is unlikely that weight gain is due solely, or even primarily, to a specific deficit. Individuals often report that they know what to do to control their weight, but are unable to motivate themselves to continue to implement these behaviors (West et al., 2011).

However, motivation for changing problem behaviors is not synonymous with motivation for participating in treatment. Those most susceptible to treatment adherence difficulties tend to be those who enter rehabilitation with little or no awareness that they have a problem that needs to be changed, those who have come as a result of being urged by others and those who have little faith in the effectiveness of interventions. They may show ambivalence about whether the problematic behavior really needs to be changed, since the perceived costs of eating may not yet outweigh the benefits (McEvoy and Nathan, 2007). Patients also differ in terms of their expectations and levels of self-efficacy for managing treatment demands.

Assessment of patients’ motivation seems, therefore, an essential prerequisite for successful weight loss therapy; for this reason many motivational approaches have been included as adjuncts into other therapeutic protocols (Waller et al., 2011).

The transtheoretical model of change (TTM;DiClemente and Prochaska, 1982; DiClemente et al., 1991; Astroth et al., 2002), for example, describes five motivational stages which clients need to advance through in order to reach a point where they can take action to change their dysfunctional behavior. This model is an integrative and comprehensive model of intentional behavior change that incorporates process-oriented variables to explain and predict how and when individuals change their own health behaviors (Sarkin et al., 2001).

The transtheoretical model has also been linked to the development of Motivational Interviewing MI; (Miller and Johnson, 2001) and its derivative Motivational Enhancement Therapy (MET; Feld et al., 2001). MI is defined as a “client-centered, directive method for enhancing intrinsic motivation to change by exploring and resolving ambivalence” (Wilson and Schlam, 2004). It is fundamentally different from other motivational approaches because motivation for change is elicited from individuals through focusing on their own ambivalence and resolving this in a way which is congruent with their own values, rather than imposed by health care providers (Miller and Rollnick, 2002). In contrast, MET aims to assist the patient with moving through the stages of motivation, by clarifying his or her own perceptions and beliefs, to reach the ultimate goal of sustained change (Feld et al., 2001). Self-Determination Theory (SDT) distinguishes between *a motivation* (lacking any intention to engage in a behavior), *intrinsic motivation* (where the behavior is engaged in for the enjoyment and satisfaction inherent in taking part), and *extrinsic motivation* (where the behavior is engaged in order to achieve outcomes that are separable from the behavior itself; Williams et al., 2004; Silva et al., 2008; Sussman et al., 2008; Jacobs et al., 2011; Verloigne et al., 2011). In fact, although intrinsic motivation is linked to greater productivity, creativity, spontaneity, cognitive flexibility, and perseverance (Deci and Ryan, 1985) human behavior is not usually intrinsically motivated (Silva et al., 2008). Deci and Ryan (1985) propose that the “quality” of motivational drive is determined not only by individual differences but also by the powerful influence of the social environment. Thus, self-motivation and treatment adherence is thereby strengthened and maintained through eliciting and acknowledging patients’ perspectives, supporting their initiatives, offering options, and providing information, whilst minimizing external pressure, and control (Williams et al., 2006; Jacobs et al., 2011). Other studies, however, highlight that there is almost no evidence that motivational interventions lead to real effects, despite being widely used either as separate therapies or integrated into other psychological treatments (Stotland et al., 2012; Waller, 2012).

Due to the negative cognitive processes (thoughts, beliefs, and images) that can psychologically “lock” people into unhealthy eating and (low) activity patterns, we have traditionally turned to cognitive behavioral therapy (CBT) as the treatment of choice for obesity and other weight issues (Wilson et al., 2010; Teufel et al., 2011; Van Dorsten and Lindley, 2011; Waller et al., 2011), even when there is strong evidence in favor of other approaches (Beutel et al., 2001; Castelnuovo et al., 2004b, 2005b, 2011c,d; Becker et al., 2007; Castelnuovo, 2010a; Kiesewetter et al., 2010; Vocks et al., 2010; Wilson et al., 2010; Teufel et al., 2011; Van Dorsten and Lindley, 2011; Waller et al., 2011; Tanofsky-Kraff, 2012, p. 130). The main goal of CBT is to change how people think and react to their problems and is widely recommended both in inpatient and outpatient settings (Melchionda et al., 2003), also in technological ones (Castelnuovo et al., 2001, 2003a,b, 2005a, 2010, 2011a,b; Castelnuovo, 2008, 2010b; Molinari et al., 2012).

More recently, Information and Telecommunication Technologies (ICT) have been utilized to address difficulties associated with of the high rate of weight regain and treatment relapse in the treatment of obesity in outpatient settings (Castelnuovo et al., 2003a; Pagliari et al., 2005).

Telecare can be used to support patients’ sense of autonomy and self-efficacy during the inpatient treatment phase as well as helping them to face daily resistances and barriers during the outpatient phase, with a range of tools such as web-sites, e-mail, chat lines (e.g., IRC, internet relay chat), videoconference, telephone, and UMTS-based mobile-phone (Castelnuovo et al., 2003a). As already indicated in several studies (Goulis et al., 2004; Rice, 2005), treatments delivered by ICT, may reduce expensive and time-consuming clinical visits and improve adherence to prescribed treatments through extensive monitoring and support (Riva et al., 2001; Castelnuovo et al., 2011d).

In the outpatient treatment phase, energy expenditure, physical activity duration, and levels as well as sleep/wake state intervals can be detected using a multisensory armband, and then transmitted online to a specific web-platform. Apart from storing data this also delivers many functions, such as questionnaires, an animated food record diary, an agenda, and a videoconference virtual room (Castelnuovo et al., 2010). During tele-sessions, clinicians (psychologists and dietitians) test outpatients’ progress, their mood, the maintenance of “good alimentary and physical activity habits,” loss/increase of weight and ask about critical moments that could lead to changes in behavior (eating and exercise; Ogden and Hills, 2008).

Since, it is the interpretation of events rather than the events *per se* that result in behaviors that lead to weight gain or loss (Ogden et al., 2009), these interpretations often need to be explicitly addressed in treatment. By utilizing videoconferencing facilities, clinical psychologists are able to deliver psychological treatments at a distance, by cognitively reconstructing dysfunctional appraisals, consolidating strategies, and abilities acquired during the inpatient phase, building self-esteem, and self-efficacy, supporting motivation to prevent relapse and providing problem-solving and crisis counseling. Telemedicine represents, therefore, a new care approach which enables us to target healthcare where it is really needed, whilst upholding the clinician-patient relationship as an important vehicle of change. Through ITC, it may therefore be possible to enhance the likelihood of success of the long-term treatment of obesity. Tele health care also allows the clinician to understand patient readiness to change, to enhance their awareness of barriers to change, to reinforce patient achievements, and to help them to prepare for relapse prevention. We propose that treatments need to move beyond the traditional cognitive approach to enhance motivation to change in the obese population. We suggest that by providing real-time technology-based interventions that enable us to monitor patients over the course of time, and by treating motivation as a behavioral rather than as an exclusively verbal-cognitive phenomenon, we will generate greater opportunities to enhance positive therapeutic change in the obese population.
